# Modified Taylor series method for solving nonlinear differential equations with mixed boundary conditions defined on finite intervals

**DOI:** 10.1186/2193-1801-3-160

**Published:** 2014-03-25

**Authors:** Hector Vazquez-Leal, Brahim Benhammouda, Uriel Antonio Filobello-Nino, Arturo Sarmiento-Reyes, Victor Manuel Jimenez-Fernandez, Antonio Marin-Hernandez, Agustin Leobardo Herrera-May, Alejandro Diaz-Sanchez, Jesus Huerta-Chua

**Affiliations:** Electronic Instrumentation and Atmospheric Sciences School, Universidad Veracruzana, Cto. Gonzalo Aguirre Beltrán S/N, 91000 Xalapa, Mexico; Higher Colleges of Technology, Abu Dhabi Men’s College, P.O. Box 25035, Abu Dhabi, United Arab Emirates; National Institute for Astrophysics, Optics and Electronics, Luis Enrique Erro No. 1, Sta. Maria, 72840 Tonantzintla, Puebla Mexico; Department of Artificial Intelligence, Universidad Veracruzana, Sebastián Camacho 5 Centro, 91000 Xalapa, Veracruz Mexico; Micro and Nanotechnology Research Center, Universidad Veracruzana, Calzada Ruiz Cortines 455, Boca del Rio, 94292 Veracruz Mexico; Facultad de Ingenieria Civil, Universidad Veracruzana, Venustiano Carranza S/N, Col. Revolucion, 93390 Poza Rica, Veracruz Mexico

**Keywords:** Taylor series method, Boundary valued problems, Shooting technique, Dirichlet conditions, Mixed boundary conditions

## Abstract

**Abstract:**

In this article, we propose the application of a modified Taylor series method (MTSM) for the approximation of nonlinear problems described on finite intervals. The issue of Taylor series method with mixed boundary conditions is circumvented using shooting constants and extra derivatives of the problem. In order to show the benefits of this proposal, three different kinds of problems are solved: three-point boundary valued problem (BVP) of third-order with a hyperbolic sine nonlinearity, two-point BVP for a second-order nonlinear differential equation with an exponential nonlinearity, and a two-point BVP for a third-order nonlinear differential equation with a radical nonlinearity. The result shows that the MTSM method is capable to generate easily computable and highly accurate approximations for nonlinear equations.

**AMS Subject Classification:**

34L30

## Introduction

Nonlinear differential equations are a popular tool to model complicated dynamical phenomenons of many branches of sciences. Unfortunately, a drawback of this approach arises when exact solutions are required but not available. On one side, pure numerical methods employed to solve nonlinear differential equations can exhibit numerical instabilities, oscillations or false equilibrium states, among others. On the other side, approximative methods are a good option when semi-analytic solutions are required. Some examples of such methods are: homotopy perturbation method (HPM) (Filobello-Nino et al. 2012a; Filobello-Nino et al. 2012b; He 1999; 2009; Khan et al. 2013; Vazquez-Leal 2012; Vazquez-Leal et al. 2012a; Vazquez-Leal et al. 2012b; Vazquez-Leal et al. 2012c), homotopy analysis method (HAM) (Hassana and El-Tawil 2011; He 2004; Tan and Abbasbandy 2008), variational iteration method (VIM) (Chang 2010; Khan et al. 2012), Taylor series method (TSM) (Barrio et al. 2011; Rodriguez and Barrio 2012; Shiraishi et al. 2011; Wazwaz 1998), Adomian decomposition method (Duan and Rach 2011; Wazwaz 1998), among others. Nevertheless, TSM highlights because of its simplicity and power; it does not require a perturbation parameter as the perturbation based techniques or trial functions as HAM or HPM does. In addition, TSM is straightforward and can be programmed using computer algebra packages like Maple or Mathematica. What is more, TSM method was conceived as a tool to solve differential equations governed by Dirichlet conditions (DC), although, mixed boundary condition (MBC) problems are important and common in several fields of physics. Therefore, in this work, we propose a modified Taylor series method (MTSM) for MBC problems that is based on the induction of shooting constants (SC) (Stoer and Bulirsch 2002). Such constants arise from two sources: 

1. The conversion of MBC to DC. This process implies the choosing of an expansion point (usually at zero) and the conversion of the MBC that are not at the expansion point to SC constants to be determined later by MTSM. The number of these constants is the same as the number of boundary conditions that are not at the expansion point.

2. Increasing the order of the original differential equation by the application of extra derivatives; as a strategy to add integration constants to solution that work as shooting constants or adjustment parameters. The number of this SC constants depends of the level of accuracy that we require from the approximate MTSM solution. For all the cases study of this work only one extra derivative is required to obtain a good fitting with respect to the exact solution, although it depends of the particular problem under study.

The aforementioned combined shooting technique of MTSM aids to circumvent the issue of TSM method with MBC. In order to show the benefits of this proposal, three nonlinear problems described with MBC on finite intervals are solved: three-point BVP for a third-order nonlinear differential equation with a hyperbolic sine nonlinearity (Duan and Rach 2011), two-point BVP for a second-order nonlinear differential equation with an exponential nonlinearity (Duan and Rach 2011; Scott and Vandevender 1975) and a two-point BVP for the third-order nonlinear differential equation with a radical nonlinearity (Duan and Rach 2011).

This paper is organized as follows. In Section ‘MTSM method’, we introduce the basic idea of MTSM method. In Section ‘Cases study’, we show the solution procedure for three nonlinear problems. Numerical simulations and a discussion about the results are provided in Section ‘Numerical simulation and discussion’. Finally, a brief conclusion is given in Section ‘Conclusion’.

## MTSM method

We consider a nonlinear differential equation expressed as 1

having as boundary condition 2

where *n* is the order of the differential equation, *N* is a general operator; *f*(*x*) is a known analytic function, *B* is a boundary operator, *Γ* is the boundary of domain *Ω*, and *∂u*/*∂η* denotes differentiation along the normal drawn outwards from *Ω*.

Firstly, we increase the order of the differential equation 3

where *k* is a constant related to the number of the desired SC constants.

Next, it is possible to express the Taylor series solution for (3) as 4

where *x*_0_ is the expansion point and derivatives *u*^(*i*)^(*x*_0_) (*i*=0,1,…) are expressed in terms of the parameters and boundary conditions of (3).

As we require to solve MBC problems, the boundary conditions not located at the chosen expansion point *x*_0_ will be replaced by shooting constants giving as result traditional DC conditions. Next, in order to obtain the coefficients of (4) (*u*^(*i*)^(*x*_0_), *i*=0,1,…), MTSM requires (I) calculate the successive derivatives of (3) and (II) evaluate each derivative using the Dirichlet conditions. Finally, in order to fulfil the boundary conditions originally replaced by the SC constants is necessary to evaluate (4) in such points; then, the resulting system of equations is solved to obtain the value of the SC constants. It is important to remark that the order of the Taylor expansion (4) is chosen in order to include all the shooting constants in the polynomial; as long as we satisfy such condition the order of the Taylor expansion can be increased to improve accuracy.

The constants due to the extra *k*-derivatives (see (3)) are applied to minimize the mean square residual (MSR) error defined as 5

where *u*_*T*_ is the approximated TSM solution (4), and [*x*_*i*_,*x*_*f*_] is the finite interval delimited by the MBC.

## Cases study

In the present section, we will solve three cases study to show the utility of the MTSM method to solve nonlinear problems. For all cases study the expansion point of TSM is at *x*_0_=0 and the derivatives were performed using Maple 17 software.

### Third-order nonlinear equation

Consider the three-point BVP for the third-order nonlinear differential equation with a hyperbolic sine nonlinearity (Duan and Rach 2011) 6

where prime denotes derivative with respect to *x* and the exact solution is unknown.

Firstly, we derive (6), resulting 7

where the boundary conditions of (6) are replaced by its Dirichlet conditions accordingly to the increased order.

Next, we resolve (7) for *u*^(*iv*)^ and perform successive derivatives, resulting 8

Now, the boundary conditions of (7) are substituted into (8), yielding 9

Finally, using (9) and the initial conditions of (7), we can formulate the sixth-order Taylor series expansion (see (4)) 10

Finally, if we substitute the boundary conditions *u*(0.25)=1 and *u*(1)=0 into (10) and solve for the shooting constants (*c*_1_ and *c*_2_), it results 11

Next, *c*_3_ is used as adjustment parameter to minimize the mean square residual (MSR) error by resolving 12

where *c*_1_ and *c*_2_ were previously substituted by (11). It is important to notice that in order to obtain a symbolic expression for *c*_3_ the hyperbolic sine was replaced by its fifth-order Taylor series.

The result of solving (12) is *c*_3_=1.1353380202 giving a minimum MSR error of 13

### Second-order nonlinear differential equation

Consider the two-point BVP second-order nonlinear differential equation with an exponential nonlinearity (Duan and Rach 2011; Scott and Vandevender 1975) 14

where prime denotes derivative with respect to *x* and exact solution is 15

Now, we derive (14), resulting 16

where the boundary conditions of (14) are replaced by its Dirichlet conditions accordingly to the increased differential equation order.

As the aforementioned procedure for first case study, we obtain the following sixth-order Taylor series 17

Finally, if we substitute the boundary condition *u*(1)=0 into (17) and solve for the shooting constant *c*_2_, it results 18

where the negative square root term was discarded because it did not minimize the mean square residual error.

Repeating the procedure for first case study (see (12)), we find that *c*_1_ = -0.4582864419 minimize the MSR error, resulting 19

where *u*_*T*_ corresponds to (17).

### Third-order nonlinear differential equation with a radical nonlinearity

Consider the two-point BVP for the third-order nonlinear differential equation with a radical nonlinearity (Duan and Rach 2011) 20

where prime denotes derivative with respect to *x* and exact solution is 21

Firstly, we derive (20), resulting 22

where the boundary conditions of (20) are replaced by its Dirichlet conditions accordingly to the increased order.

As the aforementioned procedure for first case study, we obtain the following sixth-order Taylor series 23

Finally, if we substitute the boundary condition *u*(*π*/2)=1 into (23) and solve for the shooting constant *c*_2_, it results 24

Repeating the procedure for first case study (see (12)), we find that *c*_1_ = 0.001065300514 minimizes the MSR error, resulting 25

where *u*_*T*_ corresponds to (23).

## Numerical simulation and discussion

**Figure 1:**
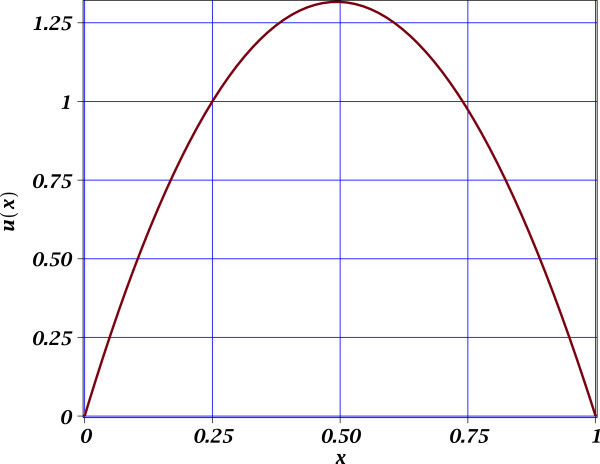
**MTSM approximation** (10) of (6). The MSR error is 0.007337036421.

**Figure 2:**
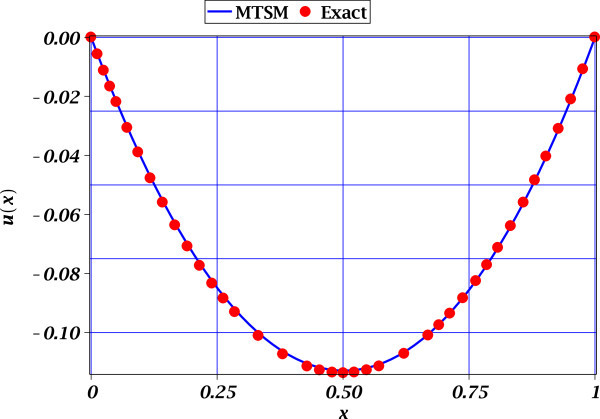
**Exact solution** (15) (solid circles) and approximate MTSM solution (17) (solid line) for (14). The MSR error is 0.0004389212651.

**Figure 3:**
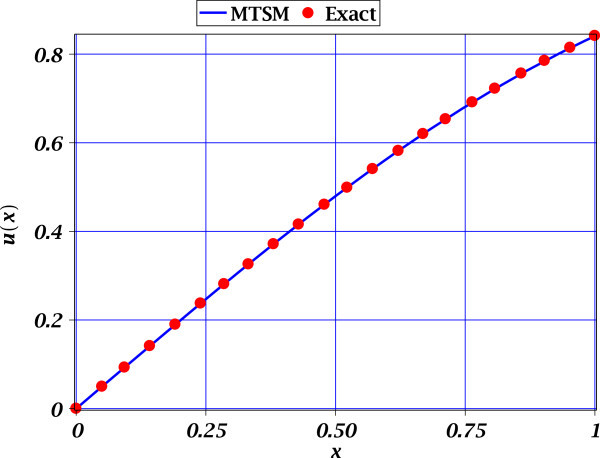
**Exact solution** (21) (solid circles) and approximate MTSM solution (23) (solid line) of (20). The MSR error is 0.0001811801833.

From Figures 1, 2 and 3, we observe the high accuracy of the proposed MTSM approximations for all cases study. For all cases study, only one extra derivative was required to obtain an acceptable low MSR error. It is important to mention that for Figures 2 and 3 the comparison is of MTSM approximations are versus the exact solutions (see (15) and (21)) depicting the accuracy of the approximations. Nonetheless, the problem (6) do not possess a known exact solution and the build-in professional numerical routines for BVP problems from Maple does failed to deal with this problem. Therefore, the only reference to know the error from exact solution is the MSR error of 0.007337036421 (see (13)), which is considered very low. This means that (10) is highly accurate.

The usefulness of coupling of a shooting method (Stoer and Bulirsch 2002) along with extra derivatives and the TSM method was exhibited by the solution of different highly nonlinear boundary value problems expressed in terms of nonlinearities such as: high order derivatives combined with hyperbolic sine, exponential and radical terms, among others. What is more, the shooting constants were used to fulfil the boundary conditions originally discarded by the artificial Dirichlet conditions. Finally, an extra derivative induced an extra shooting constant to minimize the MSR error giving as result high accurate (see (13), (19) and (25)) handy power series solutions. Finally, if users require more accurate approximated solutions, they should augment the number of SC constants (increasing *k*) to improve the potential of minimizing the MSR error (5).

In this work, we presented a modified Taylor series method to deal with nonlinear problems exhibiting mixed boundary conditions defined on finite intervals. The aforementioned procedure and results show that MTSM can obtain power series solutions using only derivatives without requiring to solve a system of differential equations or the proposal of trial functions as HPM (He 1999; 2009) or HAM (He 2004; Tan and Abbasbandy 2008) methods, or an iterative solution procedure of integrals as VIM (Chang 2010) method. In addition, MTSM is not based on the existence of a perturbation parameter (Filobello-Nino et al. 2013). Therefore, further work will address more potential applications of the proposed method to other type of problems or inclusive other type of boundary conditions as: Robin or Neumann.

## Conclusion

This work introduced the application of a modified Taylor series method (MTSM) for solving boundary value problems (BVPs) with mixed boundary conditions defined on a finite interval. We were able to obtain accurate, easy computable, handy approximations for all cases study. The shooting constants arising from the substitution of the mixed boundary conditions by Dirichlet conditions and the extra derivatives of the differential equation demonstrate - with examples - to be a powerful strategy that provides easy computable and accurate approximations. In addition, more extra derivatives can be applied to the differential equation to increase the number of shooting/adjustment constants, giving as result an enhanced convergence of the MTSM method.
